# Delayed surgical management of rectovaginal fistula: a case report highlighting challenges and lessons learned

**DOI:** 10.3389/fsurg.2023.1260355

**Published:** 2023-08-24

**Authors:** Kristina Drusany Starič, Rosario Emanuele Carlo Distefano, Giorgia Campo, Gregor Norčič

**Affiliations:** ^1^ Division of Gynaecology and Obstetrics, Department of Gynaecology, University Medical Centre Ljubljana, Ljubljana, Slovenia; ^2^ Medical Faculty, University of Ljubljana, Ljubljana, Slovenia; ^3^ Division of General Surgery and Medical Surgical Specialties, Department of Obstetrical and Gynecological Pathology, University of Catania, Catania, Italy; ^4^ Department of Abdominal Surgery, University Medical Centre Ljubljana, Ljubljana, Slovenia

**Keywords:** rectovaginal fistula, obstetric laceration, anal incontinence, delayed repair, multidisciplinary approach

## Abstract

**Background:**

Rectovaginal fistulas following an obstetric anal sphincter injury's repair are rare in developed country and their management could be challenging, particularly in cases of delayed repair. This study emphasizes the importance of accurately diagnosing and promptly repairing such fistulas for optimal patient well-being.

**Case:**

A 30-year-old patient presented with gas incontinence and a greenish discharge from the vagina, 6 months after delivering her baby. Examination revealed a small pinhole lesion on the posterior vaginal wall, and an endoanal ultrasound confirmed the presence of a rectovaginal fistula. Surgical repair was delayed for 9 months due to the patient's breastfeeding. The fistula was eventually repaired through a transrectal approach, with excision of the fistulous tract and closure of both the rectum and vagina. A laparoscopic protective ileostomy was also performed due to the delayed repair. However, a recurrence of the fistula was detected 8 months later, requiring a second repair. The patient underwent physiotherapy for the anal sphincter and achieved optimal sphincter function. After 6 months, the ileostomy was successfully closed, and the patient remained continent.

**Conclusions:**

This case highlights the importance of early recognition and prompt repair of rectovaginal fistulas following obstetric anal sphincter injury. Delayed repairs pose greater challenges and increase the risk of recurrence. Individualized surgical approaches, skilled pelvic floor repair, and a multidisciplinary approach are crucial for successful outcomes. This case underscores the need for careful planning and consideration of patient characteristics in the management of rectovaginal fistulas, aiming to achieve optimal outcomes and patient well-being.

## 1. Introduction

Obstetric anal sphincter injuries are a known complication of vaginal delivery that can lead to postpartum anal incontinence and other long-term morbidities such as perineal pain, dyspareunia, wound dehiscence, abscess formation and rectovaginal fistulas (1). They are classified into four degrees according to the depth of the injury involved (2). In fourth degree injury external and internal anal sphincter and mucosa are injured. The purpose of repairing a third- or fourth-degree laceration is to restore the continuity of both the external and internal anal sphincters (3). Recognition of anal injury with digital examination of every patient after delivery and careful end to end or overlap suturing are essential for preventing postpartum anal incontinence. When such injuries are not promptly recognized or when they are inadequately reconstructed, there is a higher risk of developing rectovaginal fistulas (3). Here we present a case of a patient that presented a rectovaginal fistula 6 months after delivery despite the fact that her fourth-degree perineal injury was adequately sutured at the time of the delivery. The location of such fistula and the delay to surgical repair due to the desire of the patient to keep breastfeeding lead to unique challenges which are therefore discussed. This case report was prepared following the CARE Guidelines (4).

## 2. Case presentation

### 2.1. Patient information

A 30-year-old female patient presented at the University Medical Centre Ljubljana in Slovenia with complaints of gas incontinence after her first delivery. She had recently given birth 6 months prior, experiencing a fourth-degree perineal injury during delivery.

The patient's chief complaints included persistent gas incontinence and a greenish discharge from the vagina. She had no significant personal and family medical history and she didn't have any prior delivery. She was a non-alcoholic and nonsmoker.

### 2.2. Clinical findings

The Jorge-Wexner score, a validated assessment tool for evaluating bowel dysfunction, was determined to be 7 for the patient, signifying a substantial impact of gas incontinence on various aspects of the patient's daily life, including social interactions, occupational activities, personal comfort, and overall well-being. The patient exhibited a normal body mass index and stable vital signs, while her abdomen was found to be palpable without any indication of rebound tenderness.

During the gynecological examination, the mucosa of the vagina and the perineal skin were observed to be properly sutured, indicating a successful reconstruction of a fourth-degree laceration. The external genitalia appeared normal in appearance, without any signs of inflammation, lesions, or abnormalities. Upon vaginal speculum examination, a small pinhole lesion was observed on the posterior wall of the vagina. This lesion exhibited a greenish discharge that was noted to emanate from it during a Valsalva maneuver. The rest of the vaginal walls were assessed and found to be intact, with no additional visible abnormalities, redness, or discharge. The cervix was visualized and appeared normal, with no visible abnormalities or lesions. Bimanual examination revealed a normal-sized and non-tender uterus, with no palpable masses or adnexal abnormalities. Despite the presence of the pinhole lesion and greenish discharge, the overall findings of the gynecological examination, including the proper healing of the reconstructed fourth-degree laceration, were within normal limits, indicating the absence of any significant gynecological pathology or abnormalities confirmed also with vaginal ultrasound, aside from the observed lesion.

### 2.3. Diagnostic assessment

As part of the diagnostic assessment, the patient underwent an endoanal ultrasound with a BK probe 2025 to evaluate the rectovaginal fistula. The ultrasound examination not only confirmed the presence of the fistula on the anterior wall, cranially to the anal sphincter, but also provided additional insights into the repair of the anal sphincter.

**Figure 1:**
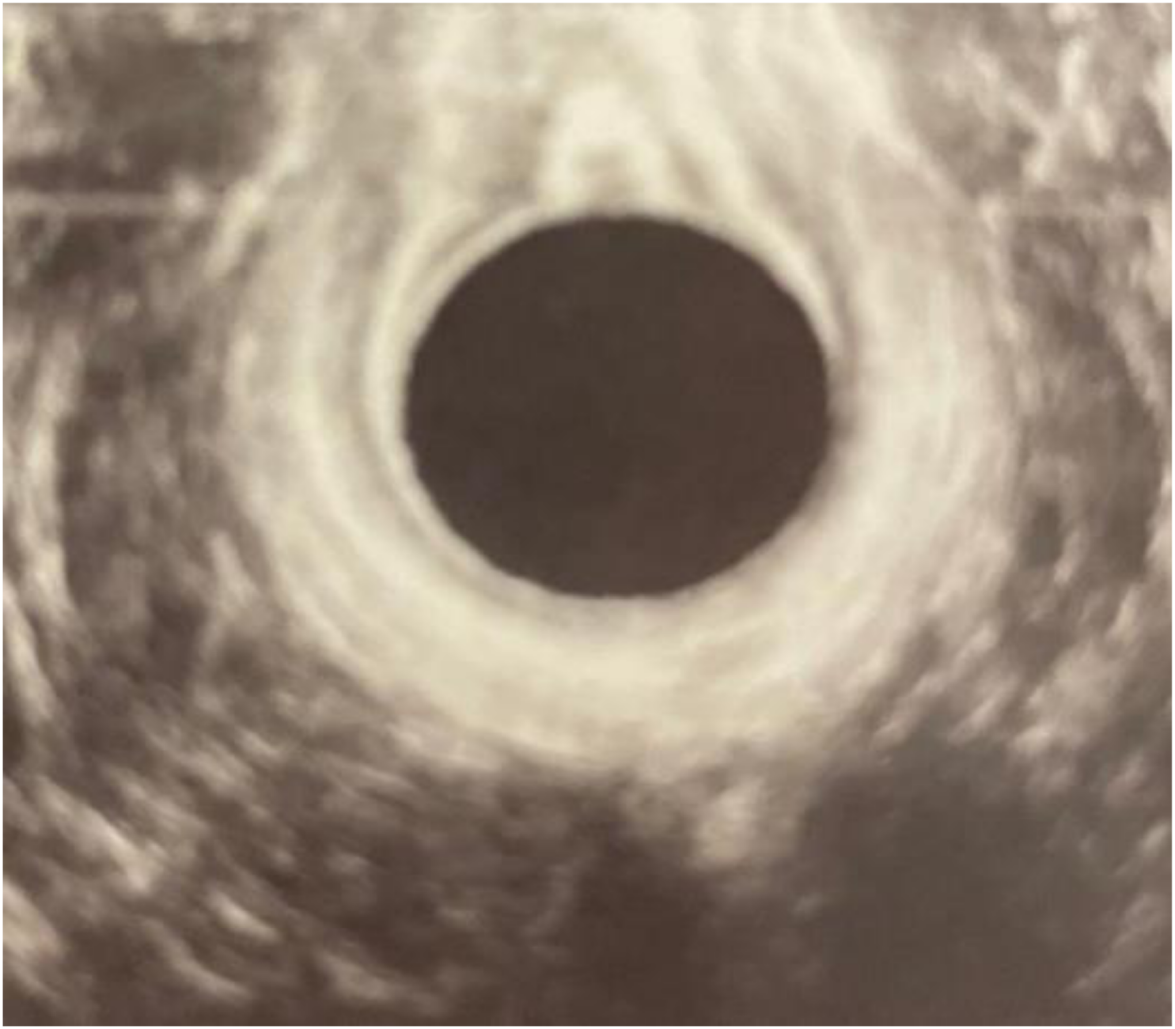
Endoanal ultrasound. The opening of the fistula on the anterior wall was shown on the ultrasound picture above the probe (black circle).

The ultrasound images revealed that the external and internal sphincters appeared optimally repaired, indicating the successful reconstruction of these critical structures during the initial repair of the obstetric anal sphincter injury (Figure 1). This finding was significant as it suggested that the previous surgical intervention had effectively restored the continuity and integrity of the anal sphincters.

**Figure 2:**
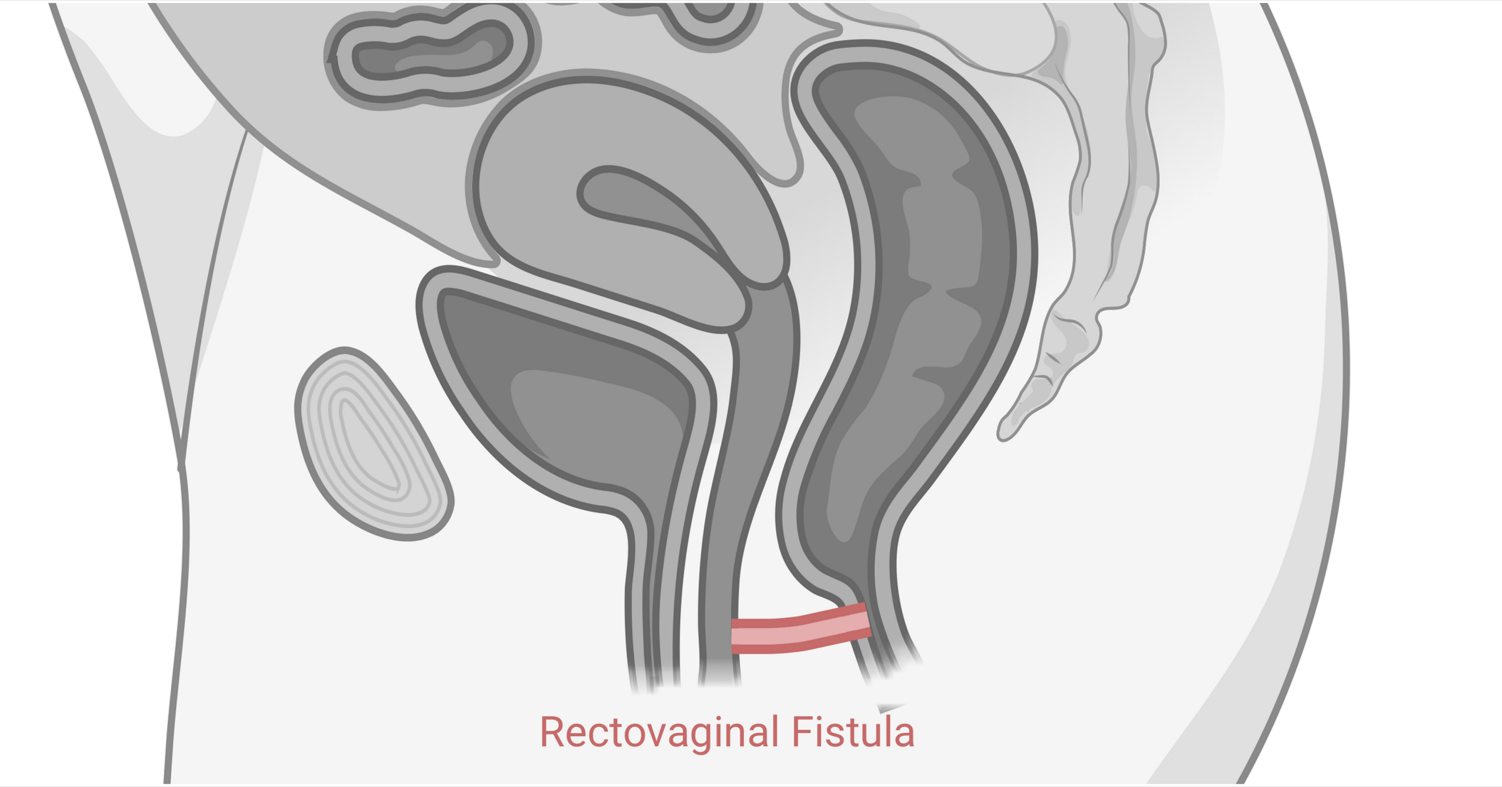
The location of rectovaginal fistula. The fistula is on the anterior wall of the rectum cranially from the anal sphincter.

In addition to the ultrasound, the diagnostic assessment also involved the administration of methylene dye on the distal third of the anal canal. This further confirmed the presence of the rectovaginal fistula, as the dye traversed the fistulous tract and became evident during the examination. The combination of the ultrasound findings and the dye confirmation provided a comprehensive understanding of the fistula's location and allowed for accurate diagnosis and treatment planning (Figure 2).

### 2.4. Therapeutic interventions

Informed consent was obtained from the patient after a detailed discussion regarding the risks and benefits of the available therapeutic options for managing the rectovaginal fistula. The patient actively participated in the decision-making process and expressed her preference for undergoing surgery. However, she requested a delay of 3 months to continue breastfeeding her child before proceeding with the surgical intervention. The patient's written consent was also obtained for the use of the photographs.

A multidisciplinary team comprising a gynecologist and a proctologist was convened to discuss the optimal approach for managing the patient's rectovaginal fistula. After thorough deliberation and weighing the advantages and potential risks, the multidisciplinary team reached a consensus to proceed with a transrectal repair approach for the rectovaginal fistula. The transrectal repair approach was deemed suitable for addressing the location and extent of the fistula, while minimizing the risk of injury to the surrounding anatomical structures, particularly the anal sphincter.

**Figure 3:**
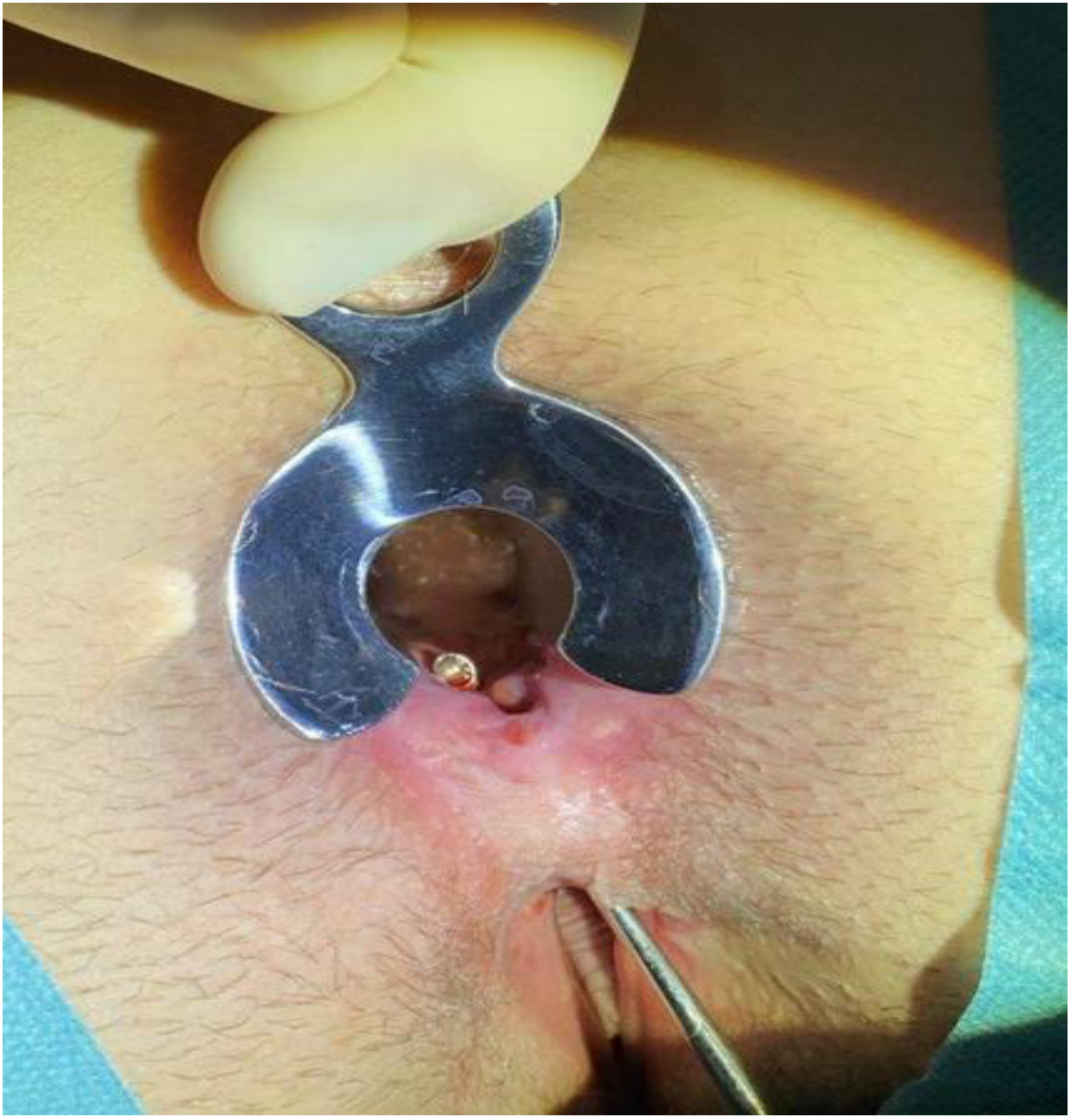
Probe position. The probe is inserted inside the rectovaginal fistula.

Under general anesthesia, the patient was positioned in a prone jack-knife position and a Lone Star Retractor System (CooperSurgical, Inc., Trumbull, USA) was attached to the anus to facilitate access to the surgical site and a probe was advanced through the rectovaginal fistula to guide the route of the fistula (Figure 3).

**Figure 4:**
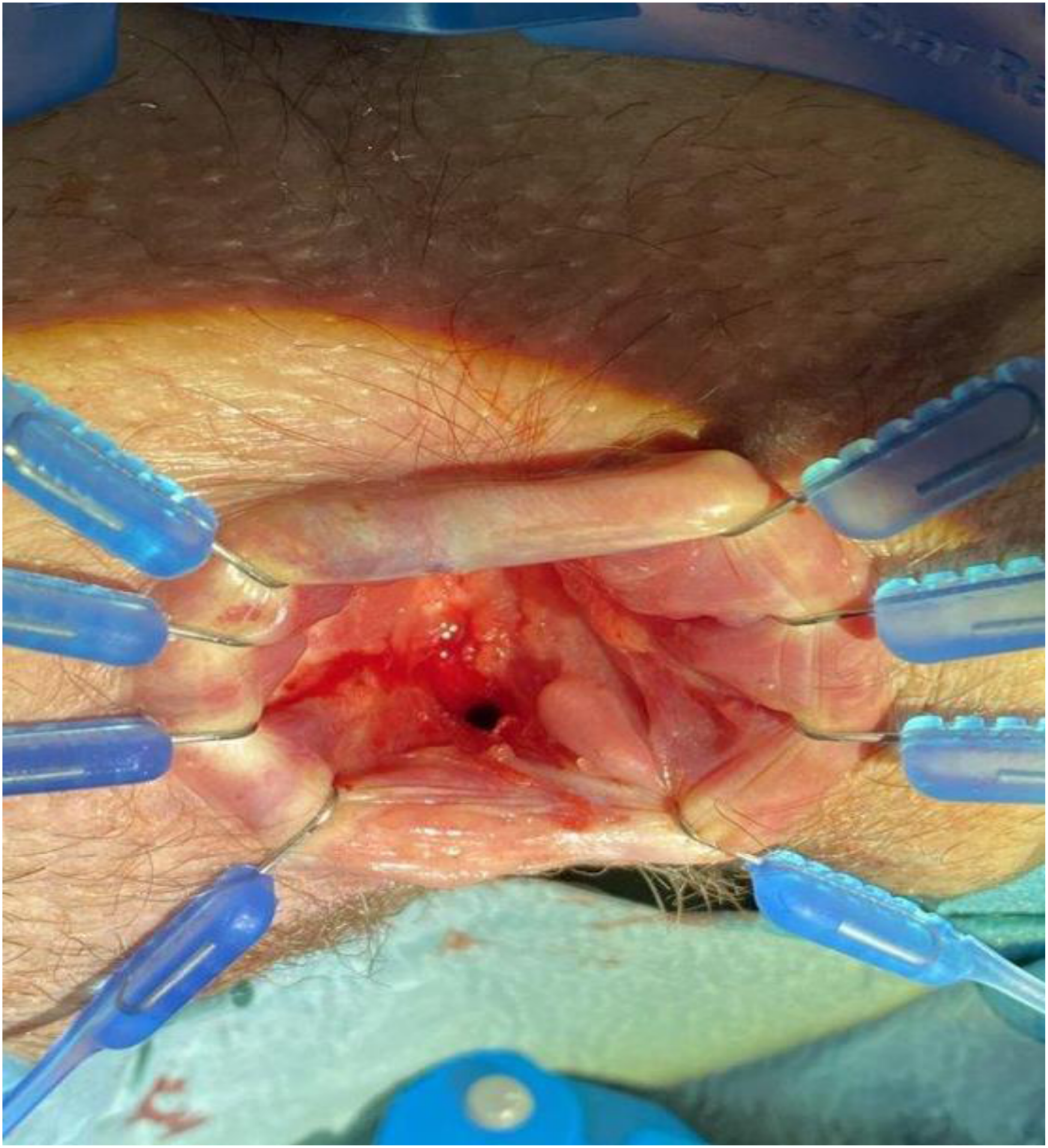
Fistula excision. The picture shows the result of the minial excision performed during the first surgery.

The next step involved the excision of the entire fistula tract that extended between the rectum and vagina. However, considering the proximity of the anal sphincter and the potential risk of compromising its integrity and subsequent anal incontinence, the width of the excision was deliberately limited (Figure 4). This cautious approach aimed to balance the complete removal of the fistula tract with the preservation of anal sphincter function.

**Figure 5:**
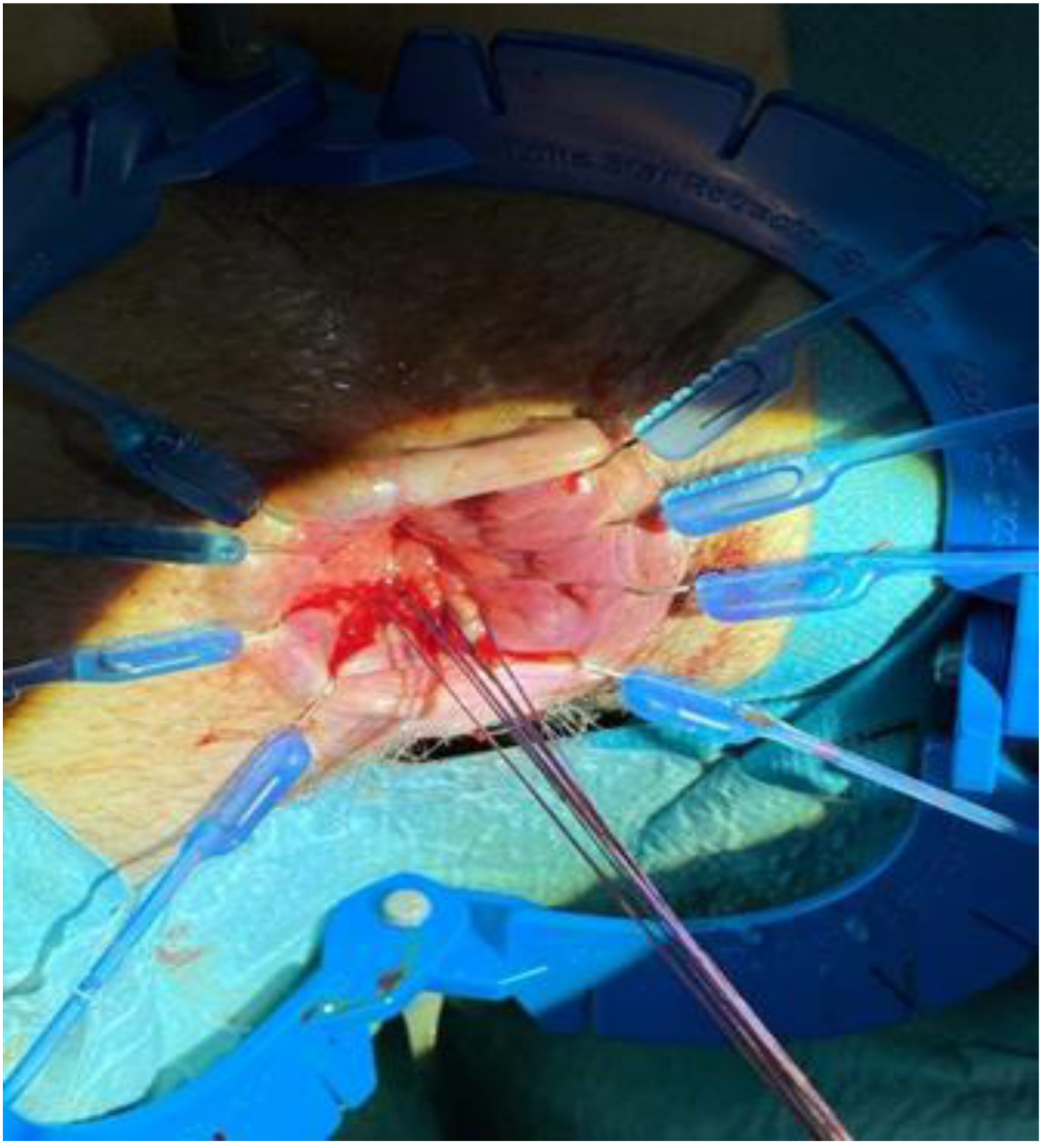
Fistula Closure. Monofilamente absorbable sutures in places before final closing of the excised fistula.

Following the excision, thorough preparation of the rectum was performed to ensure a clean and optimal surgical field. Horizontal sutures were then meticulously placed to close the rectovaginal septum using 4-0 monofilament absorbable sutures. Simultaneously, the excised fistula was closed with interrupted sutures using the same absorbable sutures (Figure 5). Throughout the procedure, utmost care was taken to ensure precise alignment and tension-free closure to optimize the chances of successful fistula closure.

Given the delayed nature of the surgery, a protective laparoscopic ileostomy was also created during the same procedure. This additional measure was undertaken to mitigate the potential risks associated with the delayed repair and provide protection to the repaired fistula site during the healing process.

Postoperatively, the patient received a course of antibiotics to prevent infection. Gentamicin was administered for 6 days, while metronidazole was prescribed for a week to address anaerobic microorganisms. These antimicrobial agents were selected based on their effectiveness in preventing surgical site infections and their suitability for the patient's specific needs.

The patient had an uneventful postoperative course and was discharged on the 6th day after surgery.

### 2.5. Follow-up and outcomes

Unfortunately, at the scheduled examination under anesthesia, 8 months after the initial surgery, a recurrence of the rectovaginal fistula was detected. In response, we performed a repeat transanal fistula repair with a more extensive excision of the fistula tract (Figures 3–5). The closure of the ileostomy was postponed in light of the recurrent fistula. The patient was administered the same antibiotic treatment as before.

After a period of 6 months, the patient remained free from any complications, and the ileostomy closure was successfully carried out. During this time, the patient underwent physiotherapy to address the function of the anal sphincter, which showed optimal improvement.

At the 6-month follow-up examination, the patient exhibited no symptoms of anal incontinence, indicating a positive outcome.

## 3. Discussion

The management of this case presented both strengths and limitations. One of the strengths was the multidisciplinary approach involving a gynecologist and proctologist, which allowed for comprehensive evaluation and decision-making. The utilization of endoanal ultrasound and methylene dye further aided in the accurate diagnosis of the rectovaginal fistula. The transrectal repair approach, although delayed due to the patient's breastfeeding preference, ultimately led to a successful closure of the fistula. Additionally, the decision to form a protective laparoscopic ileostomy during the initial repair demonstrated a proactive measure to minimize potential complications.

However, a limitation of this case was the recurrence of the fistula 8 months after the primary repair. While the subsequent surgery involved a more radical excision of the fistula tract, the possibility of recurrence highlights the challenges in managing rectovaginal fistulas, particularly in cases with delayed intervention.

Prior to delving into the existing literature, it is crucial to acknowledge that obstetric fistula represents a significant and severe childbirth complication predominantly observed in developing societies. Its prevalence is particularly pronounced in sub-Saharan Africa and South Asia, with reported rates of 1.57 and 1.60 per 1,000 women of reproductive age, respectively, in sub-Saharan Africa, and a rate of 1.20 per 1,000 in South Asia (5). In contrast, limited information is available regarding the occurrence of obstetric fistula in industrialized countries. Only one study conducted in Norway has reported an incidence of 16.3 obstetric fistula cases per 100,000 deliveries within this context (6). Despite the relatively lower incidence in industrialized countries, managing obstetric fistula in these settings presents its own set of challenges due to limited expertise and resources dedicated to addressing this condition.

Furthermore, when exploring the relevant medical literature, it becomes evident that there is no universally superior repair technique for achieving successful closure of RVFs (7–9). In a systematic review of the literature Göttgens et al. conclude that the lack of large sample sizes, robust methodology, and consistency, makes it difficult to recommend a specific approach (9). Regardless of the chosen treatment modality, the failure rate associated with RVF repair remains high. Numerous authors have reported a success rate of only 55%–60% for initial procedures, while others have reported even lower rates of 25% (7, 10). However, with subsequent procedures, the overall success rate improves to approximately 70%–90%. It is noteworthy that multiple procedures, up to a total of four, may be required to achieve successful closure of RVFs (7).

However, the classification of RVFs into simple and complex types based on factors such as size, location, etiology, and their relationship to the anal sphincter complex can guide treatment decisions (11, 12). Simple fistulas, typically smaller and more distally located, often result from trauma or infection and they are associated with better prognosis, while complex fistulas are associated with more severe conditions like inflammatory bowel disease, invasive cancer or radiation (10, 12).

The use of transanal repair approaches has shown promising outcomes in rectovaginal fistula management (13). Other surgical approaches, such as transperineal repair combined with tissue transfer procedures, have been favored by some authors for defects above the level of the sphincter complex (14). In terms of diverting stomas, their potential clinical utility lies in symptom control and support for healing. However, the clinical effectiveness of diverting stomas remains a subject of controversy due to the lack of large-scale clinical studies. Venara et al. suggest that a diverting stoma is not obligatory for managing anal sphincter injury-related rectovaginal fistulas (ARVFs) in order to enhance surgical success (7). Conversely, Corte has proposed a more aggressive surgical approach, involving the early implementation of a temporary stoma and major procedures in cases where local treatments have failed, which has demonstrated promising results in achieving high success rates (15). In our case, the subsequent transanal repair involving more extensive excision and the implementation of a diverting stoma yielded a successful outcome, suggesting the significance of a thorough surgical approach.

As mentioned in the introduction the association between severe perineal lacerations and the risk of rectovaginal fistula is well-established in the literature (2). Various factors can increase the risk of third and fourth-degree lacerations, thereby raising the risk of fistula formation (16). These factors include instrumental delivery, such as the use of forceps or spatula during delivery, and the performance of episiotomies, particularly midline episiotomies. Additionally, higher birth weights, prolonged second stage of labor, and primiparity have been linked to an increased risk of severe perineal lacerations. Based on the assessments made in this case report, the cause and effect relationship between the delayed surgery and the recurrence of the fistula cannot be definitively established. However, considering the potential implications of delayed detection and management of rectovaginal fistulas, the question of whether more clinical follow-up is warranted to better diagnose this condition needs to be addressed.

In this case report, the patient presented with a rectovaginal fistula 6 months after delivery, despite the initial adequate suturing of the fourth-degree perineal injury. Various guidelines and recommendations exist regarding the timing and nature of follow-up visits after such perineal injuries. The Royal College of Obstetricians and Gynaecologists and the American College of Obstetricians and Gynecologists recommend a follow-up appointment with a healthcare professional at around 6–12 weeks postpartum for women who have had a third or fourth-degree tear (17, 18). Whether earlier and closer follow-up visits can improve the detection and management of such condition is not clear according to our literature review.

The main “take-away” lessons from this case report are the importance of timely recognition and repair of obstetric anal sphincter injuries to prevent complications such as rectovaginal fistulas: regular postpartum assessments can help healthcare providers monitor the recovery progress, identify any signs of complications, and tailor management plans based on individual patient needs. The multidisciplinary approach involving gynecologists and proctologists can enhance decision-making and treatment outcomes (19). Additionally, individualized management and expertise in pelvic floor repairs are crucial in achieving successful outcomes in rectovaginal fistula cases. The potential challenges and risks associated with delayed surgery should be carefully considered and discussed with the breastfeeding patient, ensuring informed decision-making.

In conclusion, this case report highlights the successful management of a rectovaginal fistula through a transrectal repair approach. While there were challenges and a subsequent recurrence, the case emphasizes the significance of a multidisciplinary approach, accurate diagnosis, and timely intervention. Further research and studies are warranted to enhance our understanding and optimize the management of rectovaginal fistulas due to obstetrical perineal lacerations.

## Data Availability

The raw data supporting the conclusions of this article will be made available by the authors, without undue reservation.
